# Gaze following in pigeons increases with the number of demonstrators

**DOI:** 10.1016/j.isci.2025.112857

**Published:** 2025-06-09

**Authors:** Mathilde Delacoux, Akihiro Itahara, Fumihiro Kano

**Affiliations:** 1 Centre for the Advanced Study of Collective Behaviour, University of Konstanz, Konstanz, Germany; 2 Max Planck Institute of Animal Behavior, Konstanz, Germany; 3 International Max Planck Research School for Quantitative Behavior, Ecology and Evolution, Radolfzell, Germany; 4 Wildlife Research Center, Kyoto University, Kyoto, Japan

**Keywords:** Biological sciences, Zoology, Ornithology, Cognitive neuroscience

## Abstract

Gaze following, orienting one’s gaze in the same direction as another individual, is a key component of social attention across species, and expected to play an important role in group contexts. To investigate its collective dimension, this study tested whether the number of conspecifics providing a gaze cue influences gaze following in pigeons (*Columba livia*). Using motion capture to track fine-scale head and body movements, we presented attention-getting stimuli to subsets of pigeons (demonstrators), while others (observers) could not see them. Observer pigeons followed the gaze of demonstrators, specifically toward the target object rather than a perceptually similar distractor, and the frequency increased with the number of demonstrators. We found no evidence for nonlinear effects under our experimental conditions. In group-living species like pigeons, multiple individuals looking in the same direction may serve as a more reliable social signal, highlighting the critical role of collective context in animal social cognition.

## Highlights

• Pigeons follow the gaze of conspecifics toward a target object rather than a distractor
• Observer pigeons looked more frequently when more demonstrators were present
• The increase in gaze following responses was linear rather than saturating in our context

## Graphical abstract

**Figure:**
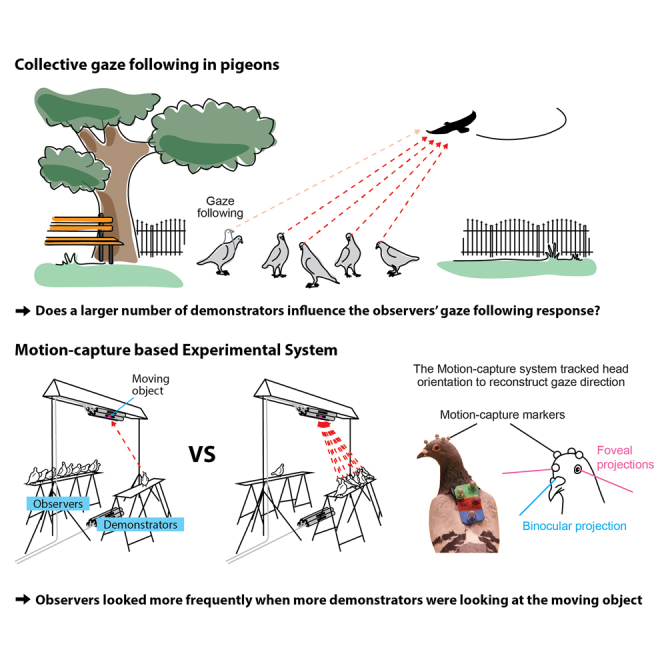

## Introduction

In many socially living animals, individual cognition is embedded within the group context and shaped by interactions among multiple individuals. Previous studies on animal collective behavior have emphasized its importance, as demonstrated by collective perception in fish schools,^1^ consensus-driven decision-making by ants, fishes^2^^,^^3^ and baboons,^4^ and cultural transmission of social information in bumblebees,^5^ great tits,^6^^,^^7^ and great apes.^8^ Despite these advancements, previous studies on animal social cognition have primarily been constrained to experimental settings with only a limited number of animals, typically dyads, rather than multiple individuals. Although the dyadic setup allows for controlled observation of both the behavior of stimulus animals and the responses of observer animals—making it particularly useful for studying the cognitive mechanisms behind individual socio-cognitive behaviors^9^^,^^10^—it overlooks key aspects of social complexity found in real-world settings.

Gaze following, defined as orienting one’s gaze in the same direction as another individual, presents an interesting research topic for exploring the effect of collective contexts on social cognition. This behavior is one of the most extensively studied socio-cognitive behaviors in animal cognition research, particularly from the perspectives of development, phylogeny, and cognitive mechanisms.^11^^,^^12^^,^^13^^,^^14^ Research has shown that gaze following is widespread across various taxa, including birds,^15^^,^^16^^,^^17^^,^^18^^,^^19^^,^^20^ primates,^21^^,^^22^^,^^23^^,^^24^ non-primate mammals,^25^^,^^26^ reptiles,^20^^,^^27^ and likely even some fish.^28^^,^^29^ While basic co-orienting of gaze may result from reflexive behavior copying, more complex pattern of behaviors, such as gaze following around barriers, likely indicate more complex cognitive processes, including perspective taking.^11^^,^^13^^,^^14^^,^^30^^,^^31^ The gaze following response is also modulated by several contextual factors, such as dominance,^32^ sex,^33^ whether the demonstrator is a conspecific or allospecific,^22^^,^^34^ facial expression,^35^^,^^36^ or communicative cues like eye contact^37^^,^^38^ (but see^39^^,^^40^). These varying levels of response are thought to engage different neural pathways: one is the subcortical route, which is fast and reflexive, while the other is the cortical pathway, which integrates social and contextual information to understand gaze in a more context-dependent manner.^41^ While basic co-orienting behavior is observed early in life, gaze following around barriers as a form of perspective-taking, typically develops later in both human and non-human animals.^42^^,^^43^^,^^44^^,^^45^

Despite the extensive research focus on its cognitive mechanisms, little is known about how gaze following occurs in a group, beyond a dyad. In humans, Milgram et al.^46^ pioneered a field experiment where a varying number of confederate demonstrators looked up at the top of a building on a street. They found that the larger the number of demonstrators exhibiting a gaze cue, the more likely passersby were to follow their gaze. Milgram et al. suspected that this response was quorum-like, based on the quadratic trend observed as a function of demonstrator number. This type of relationship, where a response is triggered only after a critical threshold is reached, is a key feature often observed in collectives such as reaching consensus in a collective decision-making process.^47^

In a similar experiment, Gallup et al.^48^ distinguished quorum from saturation by fitting a quorum response model,^47^ rather than only testing for a quadratic effect, to gaze-following data. They found that the response followed a proportional saturating pattern rather than a sharp quorum threshold. This means that an increase in the number of demonstrators led to an initial corresponding proportional increase in the observers’ looking response, but the response then gradually saturated and reached a plateau.

To our knowledge, no study has examined the effect of the number of demonstrators on gaze following in nonhuman animals. This study investigated the gaze-following responses of pigeons, where a varying number of demonstrators provided gaze cues to observers within a flock. Pigeons are well-suited to this task because they typically form relatively large flocks, where individuals are usually surrounded by multiple conspecifics (both in captivity and in the wild). In such flocks, following another’s gaze should be beneficial for key activities such as foraging and vigilance. The gaze cues provided by one versus multiple individuals might convey different meanings to an individual, as locations attended by a larger number of individuals potentially indicate the presence of more significant information.

**Figure 1:**
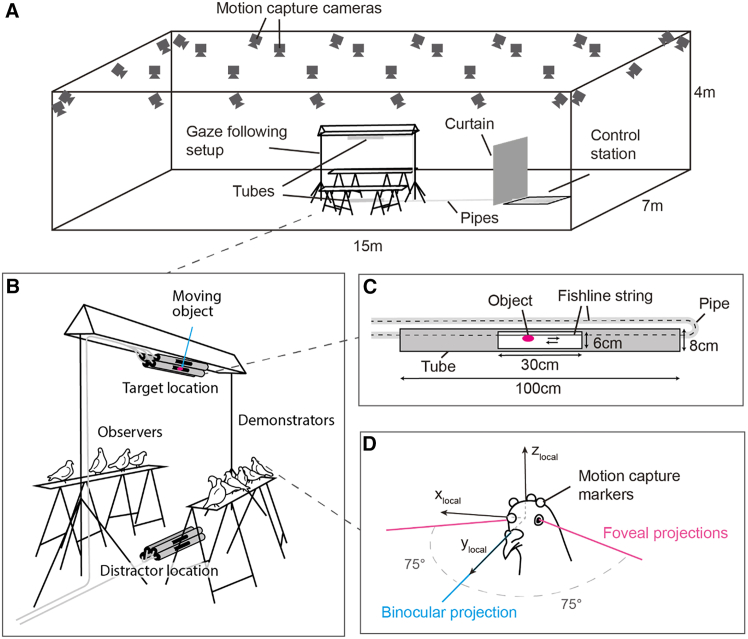
Experimental set-up (A) The experiment took place in the SMART-BARN tracking system developed by the Max Planck Institute of Animal Behavior,^51^ with the experimental setup located at its center. (B) Ten pigeons are released on the tables and can be arranged on both sides of the tubes structure by the experimenter to obtain various numbers of demonstrators and observers. Two sets of tubes were placed at the bottom and top of the structure, one containing a moving object (target location) and one not displaying anything (distractor location) to the demonstrators. (C) Small colorful objects attached to a fishline string system are presented from inside a tube, from which a window is cut, making the object visible from one side only. (D) Using the 4 motion capture markers attached to the pigeon’s head, we calculated the foveal and binocular (at horizon) projections in the head local coordinate system.

We used a recently developed motion-capture-based posture tracking system for flocks of pigeons, where all individuals’ head and body orientations were tracked with high spatiotemporal precision, allowing them to naturally flock together in an unconstrained space and engage in activities such as foraging and vigilance^49^^,^^50^^,^^51^ (Figure 1A). We used the head movement tracking data to infer the gaze reprojections of all individuals, following the method established in a previous study^50^: although eye movements were not directly tracked, this previous study showed that head orientation was a reliable proxy for looking direction in behavioral experiments. Using a structure of tubes and pipes, an experimenter could present attention-catching objects to part of the flock (demonstrators) while the other individuals (observers) could not see it (Figures 1B and 1C). Because this structure was composed of 2 sets of tubes, the object could be presented from either of the 2 sets to the demonstrators (target), while the other set would act as a perceptually similar (distractor) object to the observers. The motion-capture set-up, combined with the gaze-following experiment, is expected to capture any subtle gaze cues provided by demonstrator animals and gaze-following responses from observer animals (Figure 1D).

Although there is no direct evidence of gaze following in pigeons, several species of birds have been shown to follow the gaze of a conspecific or a human experimenter (e.g., ravens,^15^ starlings,^16^ geese,^17^ ibises,^18^ pinguins,^19^ tinamous, emus, rheas, and jungle fowls^20^). We thus expected that pigeons also follow the gaze of at least their conspecifics.

This study aimed to answer four questions: (1) whether pigeons exhibit gaze-following responses, (2) whether a larger number of demonstrators enhance the observers’ gaze-following responses, and (3) if so, whether the increase in response follows a linear, quorum, or saturation pattern. As a secondary question, we also asked (4) whether pigeons could distinguish the gaze target of the demonstrators from a distractor object (a perceptually equivalent object). This last question has not been addressed in previous studies on bird gaze following, largely due to the lack of a fine-scale tracking system. This question is particularly interesting because many bird species have two or more types of “gaze”. In pigeons, two laterally projecting foveas are used when attending to distant objects, while the binocular field (region were both monocular fields overlap, ∼20° in pigeons when their eyes are in resting position^52^) is engaged when pecking at seeds at close range,^50^ perching^53^ or tracking slowly moving objects.^54^ Thus, it remains unclear whether observer pigeons can identify the gaze target of demonstrators when both target and distractor objects are present, given the multiple possibilities for gaze direction.

## Results

### Gaze following by pigeons

To address question (1) whether pigeons exhibit gaze-following responses, we tested whether observers showed an increased likelihood of looking and a larger number of looks at the target location in the test condition compared to the control condition.

We analyzed the data using generalized linear mixed models (GLMMs). The primary response variables were (1) the likelihood of an observer looking at the target location (analyzed using binomial models) and (2) the number of looks at the target location per presentation (analyzed using Poisson models). The main predictor was the condition (test or control), with control variables included (see STAR Methods).

**Figure 2:**
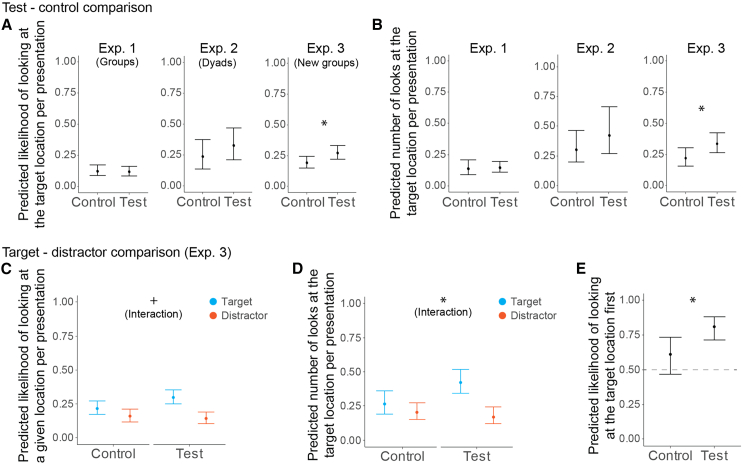
Gaze following results of pigeons (A and B) Test-Control comparison: standard way of testing gaze following in dyadic setups. The graphs show the looking response (A) likelihood of looking and (B) number of looks as a function of condition. (C–E) Comparison of the response toward the target vs. distractor location in the test and the control conditions in the Exp. 3, to test if the pigeons responded specifically more to the target compared to the distractor location in the test setup. The graphs show the looking response (C) likelihood of looking, (D) number of looks and (E) likelihood of looking at the target first as a function of condition for the target and distractor locations. For (C and D), symbols denotates the significance of the interaction condition∗location. For all graphs, confidence intervals (CI) were calculated using 10 000 simulations of the model parameters and represents the range between the 2.5th and 97.5th percentiles of the simulated predictions. *p* = 0.1 > + > 0.05 > ∗ >0.01 > ∗∗ >0.001 > ∗∗∗.

In the test condition of Exp. 1 (*n* = 24) a varying number of pigeons from a flock of ten could see a moving object, while the others could not. In the control condition, none of the pigeons could see the moving object, as all were moved to the side where the object was not visible. This control condition established a baseline observation and controlled for a potential effect of sound from the moving object. In Exp. 1, we did not find a significant effect of condition on neither the observers’ likelihood of looking (*χ*^*2*^(1) = 0.113, *β* = −0.066, *p* = 0.736; Figure 2A; see Table S2 for more details on model results) nor the observers’ number of looks (*χ*^*2*^(1) = 0.077, *β* = 0.061, *p* = 0.782; Figure 2B).

We therefore conducted Exp. 2, testing the same pigeons, to confirm gaze-following responses in a more conventional dyadic setup. Additionally, we modified the control condition, by presenting the object from a tube whose window was occluded, rather than moving the pigeons to the side where they could not see the object (see STAR Methods).

Although we found some indications of gaze following in this experiment, the difference between test and control conditions did not reach statistical significance (likelihood of looking: *χ*^*2*^(1) = 1.848, *β* = 0.443, *p* = 0.174; Figure 2A; see Table S2 for more details; observers’ number of looks: *χ*^*2*^(1) = 2.006, *β* = 0.335, *p* = 0.157; Figure 2B). In a follow-up test (Exp. 2 follow-up), we used a human demonstrator instead of a conspecific demonstrator. While the latter might provide more biologically meaningful information, the human demonstrator could give more controlled and standardized cues. However, we did not find a significant effect of the condition neither on the likelihood of looking at the target (χ2(1) = 0.15, *p* = 0.70; see Figure S2 and Table S2), nor the number of looks at the target (χ2(1) = −0.04, *p* = 1).

We further improved the study design in Exp. 3, testing a larger new group of pigeons (*n* = 46) while performing fewer trials per individual to prevent habituation of the demonstrators. Additionally, we modified the control condition by presenting the object from an occluded tube instead of moving the pigeons (as in Exp. 2) and improved counterbalancing between test and control conditions (see STAR Methods). In Exp. 3, we found a significant effect of condition on both the likelihood of looking (*χ*^*2*^(1) = 4.964, *β* = 0.448, *p* = 0.026; Figure 2A) and the number of looks by the observers (*χ*^*2*^(1) = 6.574, *β* = 0.425, *p* = 0.010; Figure 2B). Pigeons were more likely to look at the target and did so more frequently in the test condition compared to the control condition.

To address question (4) whether pigeons could distinguish the demonstrators’ gaze target from a distractor object, we examined the likelihood of looking at the target location first using the same model structure. We found that, in the test condition, the first look was significantly more likely to be directed toward the target location than the distractor location compared to the control condition (*χ*^*2*^(1) = 5.917, *β* = 1.005, *p* = 0.015; Figure 2E). Additionally, we tested if the demonstrators’ cues (test condition) had a different effect on the observer’s response toward the target compared to the distractor, by including an interaction term between condition (test, control) and object type (target, distractor) in the model. For the number of looks, we found a significant effect (*χ*^*2*^(1) = 5.287, *β* = −0.644, *p* = 0.021; Figure 2D). This result indicates that observer pigeons looked more at the target than the distractor in the test condition compared to the control condition. A similar trend was observed for the likelihood of looking, but it did not reach statistical significance (*χ*^*2*^(1) = 3.556, *β* = −0.565, *p* = 0.059; Figure 2C).

### Collective effect on gaze following

To address question (2), whether a larger number of demonstrators enhances observers’ gaze-following responses, we tested the effect of the “actual number of demonstrators” to assess a potential collective influence on gaze following in GLMM. The “actual number of demonstrators” represents the number of pigeons on the demonstrator side that provided a gaze cue—i.e., those that looked at the target at least once during the presentation. We used data from the test conditions in Exp. 1 and Exp. 3, first analyzing each experiment separately and then conducting a combined analysis to ensure sufficient data for statistical testing (the experimental design for the test condition was identical between Exp. 1 and Exp. 3).

**Figure 3:**
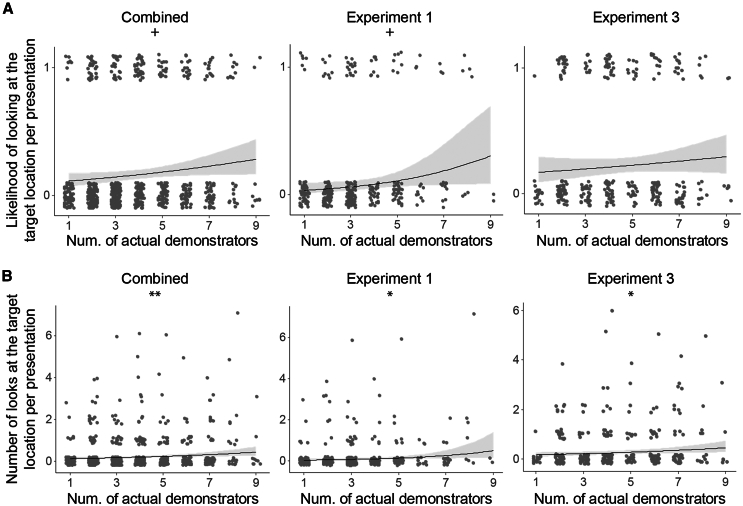
Collective effect on gaze following (A and B) Looking response (A) likelihood of looking and (B) number of looks as a function of the number of actual demonstrators for the combined dataset as well as for Exp. 1 and 3 separately. For all depicted results, regression lines were determined with other variables held constant, set to their mean values and the ribbon shows the 95% confidence interval (based on 10 000 simulations of the parameters). Individual datapoints are represented with vertical and horizontal jitter for better visualization. *p* = 0.1 > + > 0.05 > ∗ >0.01 > ∗∗ >0.001 > ∗∗∗.

In Exp. 1, we found a significant effect of the number of actual demonstrators on the observers’ number of looks (*χ*^*2*^(1) = 6.028, *β* = 0.583, *p* = 0.014; Figure 3B). A similar trend was observed for the observers’ likelihood of looking at the target (*χ*^*2*^(1) = 3.215, *β* = 0.567, *p* = 0.073; Figure 3A; see Table S2 for more details), although this effect was not statistically significant. In Exp. 3, we also found a significant effect of the number of actual demonstrators on the number of looks by the observers (*χ*^*2*^(1) = 4.535, *β* = 0.263, *p* = 0.033; Figure 3B). However, the effect on the likelihood of looking was not significant (*χ*^*2*^(1) = 0.213, *β* = 0.183, *p* = 0.685; Figure 3A). In the combined dataset for Exp. 1 and Exp. 3, we found a significant effect of the number of actual demonstrators on the number of looks (*χ*^*2*^(1) = 8.902, *β* = 0.324, *p* = 0.003; Figure 3B). A similar trend was observed for the likelihood of looking, though it did not reach statistical significance (*χ*^*2*^(1) = 3.623, *β* = 0.285, *p* = 0.057; Figure 3A). Moreover, the positive *β* estimates indicate an increase in the observers' response in with the number of demonstrators in all models.

To determine whether the configuration of pigeon locations in the test condition (specifically, the number of demonstrators present rather than those actually looking at the target) predicted the observers’ response, we replaced the “number of actual demonstrators” variable with the “number of demonstrators” in the same model. Overall, this was a weaker predictor of the observers’ response (likelihood of looking: *χ*^*2*^(1) = 2.464, *β* = 0.221, *p* = 0.117; number of looks: *χ*^*2*^(1) = 3.893, *β* = 0.207, *p* = 0.048; see Table S2 for more details on model results). Additionally, the models using “number of demonstrators” had larger AIC values, suggesting lower performance compared to the original models (likelihood of looking: AIC = 685.9 vs. 690.5; number of looks: AIC = 1050.9 vs. 1058.4).

Finally, to address question (3) whether the increase in response follows a linear, quorum, or saturation pattern, we first tested for non-linearity by adding a quadratic effect of the number of actual demonstrators to the same model using the combined dataset. This aimed to test for a change in the slope, indicating a potential saturation of the observed effect. The quadratic effect was not significant for either the likelihood of looking (*χ*^*2*^(1) = 1.504, *β* = −0.646, *p* = 0.220; see Table S2 for more details) or the number of looks (*χ*^*2*^(1) = 1.478, *β* = −0.556, *p* = 0.224). Additionally, the linear model consistently outperformed the quadratic model, as indicated by smaller AIC values (likelihood of looking: AIC = 685.9 vs. 697.0; number of looks: AIC = 1050.9 vs. 1057.0).

## Discussion

Overall, our results supported that (1) pigeons follow the gaze of conspecifics, (2) a larger number of demonstrator pigeons increased the observer pigeons’ gaze following responses, (3) this increase was linear, and (4) observer pigeons distinguished the demonstrators’ gaze target, whether it was above or down.

### Gaze following in pigeons

Our results align with previous studies that demonstrate gaze following of conspecifics and human demonstrators across species, including several bird species (ravens,^15^ starlings,^16^ geese,^17^ ibises,^18^ pinguins,^19^ tinamous, emus, rheas, and jungle fowls^20^). However, it is worth noting that the gaze-following responses of our pigeons appeared relatively weak compared to the performance of other bird species in earlier studies,^16^^,^^18^^,^^20^^,^^45^ although other studies have reported similar rates.^15^^,^^17^^,^^19^ The reason for the relatively weak gaze-following responses in our pigeons remains unclear. It may be that pigeons do not follow the gaze of conspecifics as strongly as other species. Alternatively, various contextual factors —such as the pigeons’ experience and subtle environmental influences— may have affected the overall results, warranting further research on gaze following across species.

Moreover, while Exp. 3 demonstrated gaze following in pigeons, Exp. 1 and 2 did not show a significant effect. In Exp. 1, this absence could be explained by the habituation of the demonstrators to the moving objects, leading to weaker observer responses (see Figure S3). Exp. 2, which closely mimicked conventional gaze-following setups from previous studies, ensured that the demonstrator pigeon looked at the target object at least once. However, although some trends in the predicted direction were observed, it still failed to show clear evidence of gaze following (possibly reflecting species or contextual limitations). Exp. 3, however, yielded significant results, likely due to the use of a larger group of new pigeons, which allowed fewer trials and reduced demonstrator habituation, along with improved control conditions.

Interestingly, in Exp. 3, we found that pigeons looked at the target more often than at the distractor, indicating that they specifically oriented their gaze toward the target. This suggests that the observer pigeons could distinguish at least whether the demonstrator pigeons were looking up or down. On these occasions, the demonstrator pigeons typically used one of their laterally projecting foveas to view the target object (92% of the looks toward the target, against 8% for binocular vision at horizon). In our table setups, while one fovea was directed at the target (in the direction of the observer pigeons), the other fovea was directed away from the set-up (both target and distractor). Therefore, it is likely that the observer pigeons not only responded to the demonstrators’ head tilts but also specifically followed the gaze of either eye that was directed toward the target.

### Collective effect on gaze following

Our results show that an increased number of demonstrators looking at the target location enhances the observers’ responses. This is largely consistent with human responses in a street context, although we did not observe the saturation effects reported in previous studies.^48^ To our knowledge, this study is the first to demonstrate a collective effect in non-human animals. Moreover, the increase in observers’ responses is better explained by the number of demonstrators giving a gaze cue toward the target than the number of demonstrators on the other table. This ensured that it was the demonstrators’ looking behavior, in contrast to the spatial configuration of the demonstrators and observers that affected the observers’ gaze following.

The absence of a nonlinear effect in our experiments is likely due to procedural factors, as the maximum number of demonstrators used—nine—was relatively moderate. A saturation effect may still emerge with a larger number of demonstrators. Additionally, as mentioned, the pigeons’ gaze-following response was relatively weak. Nevertheless, our results clearly demonstrated that the collective context significantly influences gaze following in pigeons. This finding is further supported by the fact that our dyadic setup failed to produce evidence of gaze following. One possible explanation is that in group-living species like pigeons, a single individual looking may not be enough to elicit a gaze-following response from others, as the likelihood of any one individual looking at any given moment is high. By contrast, multiple individuals looking in the same direction likely serves as a more reliable indicator of something important in the environment.

An alternative explanation is that an observer is more likely to follow a larger number of demonstrators simply due to increased opportunity; namely, with more demonstrators looking, the observer is more likely to notice at least one gaze cue. However, we designed our table setup so that the observer pigeons could see all demonstrators simultaneously at least with their peripheral vision, with the demonstrators positioned roughly equidistantly from the observers and minimal occlusion between them. Additionally, despite using a dyadic setup and ensuring the demonstrator’s gaze, Exp. 2 failed to reveal a significant effect. This could indicate that one demonstrator is not sufficient to elicit a gaze following response in pigeons.

Overall, we found gaze following in pigeons depends on the number of demonstrators. Quantity or number discrimination is a common cognitive skill present across various species,^55^^,^^56^^,^^57^^,^^58^ including pigeons.^59^^,^^60^^,^^61^ Our results suggest that, in addition to other contextual factors—such as dominance in monkeys,^32^ gender in humans,^33^ model species in apes,^22^^,^^34^ facial expressions in both monkeys and humans,^35^^,^^36^ communicative cues in both dogs and humans^37^^,^^38^ —the number of demonstrators may be another factor that modulates gaze following in both humans^48^ and pigeons. Further studies are needed to examine this factor in other species.

### Limitations of the study

While we found consistent evidence for gaze following and an effect of the number of demonstrators, the overall response of the pigeons was relatively weak. The reasons for this are unclear but may relate to aspects of the experimental design or setup. For example, repeated trials may have led to habituation among the demonstrators, reducing the salience of their gaze cues over time. Combined with the relatively small number of demonstrators used, this may have limited our ability to detect more nuanced patterns, such as saturation effects or quorum-like responses. Future research could build on this work by examining the collective dynamics of gaze following and other social cognitive processes across a broader range of species and in more naturalistic group settings. Such efforts are particularly important because these behaviors likely evolved to function in group contexts that often involve interactions beyond dyads—a perspective frequently overlooked in animal cognition studies.

## Resource availability

### Lead contact

Requests for further information and resources should be directed to and will be fulfilled by the lead contact, Mathilde Delacoux (mdelacoux@ab.mpg.de).

### Material availability

This study did not generate new unique reagents.

### Data and code availability

• Data: The data used for the analysis have been deposited on an OSF repository (https://osf.io/bc9gz/; https://doi.org/10.17605/OSF.IO/BC9GZ). They are publicly available as of the date of publication.
• Code: The code used for the analysis have been deposited on an OSF repository (https://osf.io/bc9gz/; https://doi.org/10.17605/OSF.IO/BC9GZ; see also supplementary code from^49^: https://osf.io/d682s/; https://doi.org/10.17605/OSF.IO/D682S). They are publicly available as of the date of publication.
• Additional information: Any additional information required to reanalyze the data reported in this paper is available from the lead contact upon request.

## Acknowledgments

We would like to thank Drs. Mate Nagy, Dora Biro, Oliver Deussen, and Iain D. Couzin, as well as all the other members of the Max Planck Institute of Animal Behavior (MPI-AB) and Center for the Advanced Study of Collective Behavior (CASCB), for their insightful feedback and support. Our thanks also go to Mathias Günther for his technical assistance with the use of the facilities. Lastly, we also want to thank Drs. Inge Müller, Daniel Zuniga, and the caretakers, for their dedicated care of the pigeons. This study was funded by MPI-AB, the DFG Cluster of Excellence 2117 CASCB (ID: 422037984), and the CASCB BigChunk projects (ID: L21-07), as well as the Grant for Overseas Research by the Division of Graduate Studies, Kyoto University.

## Author contributions

M.D., A.I., and F.K. conceived the design and setup; M.D. and F.K. performed the experiments; M.D. and F.K. analyzed the data; M.D. and F.K. wrote the manuscript’s first draft; M.D., A.I., and F.K., contributed to the final version of the manuscript.

## Declaration of interests

The authors declare having no competing interests.

## Declaration of generative AI and AI-assisted technologies in the writing process

During the preparation of this work, the authors used ChatGPT^62^ in order to correct grammar and refine readability. After using this tool, the authors reviewed and edited the content as needed and take full responsibility for the content of the publication.

## STAR★Methods

### Key resources table

**Table:** 

REAGENT or RESOURCE	SOURCE	IDENTIFIER
**Deposited data**		

Raw and analyzed data	This paper	OSF: https://osf.io/bc9gz/ https://doi.org/10.17605/OSF.IO/BC9GZ

**Experimental models: Organisms/strains**		

Pigeons *(Columba livia)* wild type	Duivenkweekcentrum Limburg (The Netherlands)	RRID: NCBITaxon_8932

**Software and algorithms**		

Processing of the Motion-Capture data (in MATLAB and Python)	Delacoux and Kano^63^	OSF: https://osf.io/d682s https://doi.org/10.17605/OSF.IO/D682S
Quantification of looking (in MATLAB)	This paper	OSF: https://osf.io/bc9gz/ https://doi.org/10.17605/OSF.IO/BC9GZ
Statistical analysis (in R)	This paper	OSF: https://osf.io/bc9gz/ https://doi.org/10.17605/OSF.IO/BC9GZ

### Experimental model and study participant details

A total of 70 juvenile pigeons *(Columba livia)* were tested in this study. The first and second experiments tested 24 1-year-old pigeons (9 males and 15 females) while the third experiment tested 46 less than 1-year-old pigeons (15 males and 31 females). They were housed in an outdoor aviary 2 × 4 × 2m at the Max-Planck Institute of Animal Behavior, Radolfzell, Germany. They had *ad libidum* access to water and grit and were fed once a day with a mix of seeds.

Animal experiments in this study were performed under the licenses 35–9185.81/G-19/107 and 35–9185.81/G-22/100 granted by the Regierungspräsidium Freiburg, Abteilung Landwirtschaft, Ländlicher Raum, Veterinär-und Lebensmittelwesen, animal ethics authorities of Baden-Württemberg, Germany. No animal was injured or killed during the tests and handling was kept minimal to reduce stress. Outside of experiments, the pigeons were socially housed in their home aviary, provided with perches and nesting structures, and checked daily for health state. After the daily experiment was completed, the markers were removed from the pigeons’ heads and backpacks, and they were brought back to their loft.

### Method details

#### Experimental design and rationales

This study consisted of 3 experiments aimed at demonstrating gaze following in pigeons by progressively refining the experimental setup throughout the experiments. The design of Exp. 2 followed a typical dyadic setup, while Exp. 1 and 3 were performed in a group setup to additionally investigate an effect of the number of demonstrators. These 3 experiments were analyzed separately, but to ensure sufficient data for analysis, we also combined the results of Exp. 1 and 3 (only for the test data, whose design is identical in both experiments). Exp. 1 was performed with a first batch of pigeons (*n* = 24). In the test condition, a varying number of pigeons from a flock of ten could see a moving object while the others could not see. In the control condition, none of the pigeons could see the moving object, thereby establishing a baseline observation as well as controlling for the potential effect of sound from the moving object. In Exp. 1, while we observed an effect of the number of demonstrators on the observer’s behavior, there was no significant difference between test and control conditions. Exp. 2 tested the same pigeons and aimed to confirm the gaze-following responses in a more conventional setting, in a dyadic setup with either a conspecific, or a human as a demonstrator (respectively Exp. 2 and Exp. 2 follow-up). In addition, we modified the control condition. Although we found some indication of gaze following in this experiment, the difference between test and control conditions did not reach statistical significance. Given that we expect a collective effect on gaze following, Exp. 3 tested a larger number of new pigeons (*n* = 46), while performing fewer trial for each individual to avoid habituation of the demonstrators, and adopting the improved control design.

#### Experimental set-ups

##### Motion-capture system

The experiments were conducted in the SMART-BARN^51^ (Figure 1A). The setup included a motion-capture system with 32 infrared cameras covering a 15 × 7 × 4 meter volume. At the center, an inverted U-shaped structure (3w x 2h m (w = width, h = height)) held 8 opaque tubes (4 on top, 4 on the ground, Figure 1B), each containing a small colorful object (4–5 cm diameter, including biologically relevant stimuli such as grains or animal-shaped objects) attached to a string control system. Each tube had a small window from which the object is visible only from one side Figure 1C. A set of pipes was leading the strings coming out of the tube structure to a control station, hidden from the pigeons’ sight by a curtain, where the experimenter can control the objects movements. Pigeons were placed on two wooden tables (210L x 22w x 75h cm; l = length) positioned parallel to the structure, where they could either see ("demonstrators") or could not see ("observers") the moving objects through the window. During experiments, the objects were moved back and forth in either the top or bottom tubes. The location presenting the objects is referred as the "target" location while the other location not presenting the objects is referred as the "distractor" location. After habituation to the structure and tables, pigeons naturally preferred standing on the tables and no particular training was required. Prior to the daily experiment, the motion capture cameras were calibrated using a standard procedure with Active Wand (VICON; calibration with 2000 frames per camera).

##### Marker attachment and eye-beak calibration

Four motion capture markers (6.4mm diameter, OptiTrack) were placed on the head of the pigeons for the tracking of head orientations. In addition, a Styrofoam plate assorted with a unique combination of 4 markers (9mm diameter) was attached to their back for identification. The head of the pigeons was recorded from 4 different angles with synchronized webcams. This procedure (further detailed in^50^^,^^51^) allowed for the reconstruction of the eyes and beak tip positions relative to the head markers.

#### Experimental procedures

##### General

Each pigeon experienced no more than a trial per day. In the experiment, the pigeons were released on tables on either side of the structure. Within each trial, they were exposed to a succession of object presentations. In each object presentation, the experimenter, hidden behind a curtain, pulled fish lines attached to the object back-and-forth twice for about 24 s. Before and after each object presentation, the object was placed in the occluded sections of the tubes. The interval between object presentations was varied randomly (min 20 s, mean 54 s), to prevent learning of the object appearances by the pigeons. The ID of all pigeons was tracked at all times.

##### Group set-up (Exp 1 and 3)

Ten pigeons were released on the tables. The pigeons' locations were shuffled between presentations by the experimenter gently approaching one of the tables, triggering flight responses to the other table, ensuring that all pigeons experienced different numbers of demonstrators. In the test condition, identical in both Exp. 1 and 3, demonstrators saw the moving object and could provide gaze cues to observers. However, Exp. 1 and Exp. 3 differ in the control condition and in the number of trials and object presentations. In the control condition of Exp. 1, all pigeons were moved to one side (observers only), and objects were presented on the opposite side (no demonstrator; Figure S1). In the control condition of Exp. 3 (as well as Exp. 2), the object was presented in an occluded tube (no window) while keeping the numbers and identities of demonstrator and observer pigeons as similar as possible between conditions (Figure S1). By performing the test and control conditions in two successive trials, this allowed for a better pairwise comparison. For the number of trials, Exp. 1 had 8 test and 4 control object presentations (we required more test presentations than controls, as only observers provided data in tests, while all 10 pigeons contributed data in controls) in pseudo-counterbalanced order across trials (either TTTTCCTTTTCC or CCTTTTCCTTTT; with T = one test presentation and C = one control presentation), and each pigeon underwent 4–6 trials. Exp. 3 had 8 test and 8 control object presentations (only pigeons standing on the “observer” table contributed to both the test and control data) in a randomly counterbalanced order across trials (e.g., TC-CT-TC-TC-CT-TC-CT-CT), and each pigeon underwent 2–3 trials (fewer trials to avoid habituation).

##### Dyadic set-up (Exp 2)

In Exp. 2, we presented objects from the same tube structure setup, only on the top tubes (no bottom tube). Two pigeons were placed on opposite sides of a small table and separated by a thin net. Each trial consisted of two test presentations (with each pigeon acting once as a demonstrator and once as an observer) and one control presentation, where the object was in an occluded tube and no pigeon could see it. The sequence was randomized, and each pigeon experienced six trials on different days with different pairs (one trial per day). As a follow-up, we also performed an experiment with the identical design except that the demonstrator is a human experimenter as in some previous studies (ravens,^15^ parrots,^64^ bonobos,^22^ dogs,^26^ jackdaws^65^) to present longer and more standardized gaze cues. An experimenter stood on one side of the structure and was separated from the pigeon by a thin net. The gaze cues were then exhibited in a standard manner (attracting the pigeon’s attention, giving a 5 s gaze cue, looking slightly down for 10 s, followed by a 20 s break between 2 presentations). The gaze cue was either looking up at the structure (test) or looking behind the pigeon (control). One trial consisted of 3 test and 3 control presentations shuffled in random sequences (each pigeon experiencing 2 trials).

### Quantification and statistical analysis

#### Mo-cap data processing

The details of the pipelines used to process the motion capture data can be found in Delacoux & Kano^49^ and the code is available in Delacoux & Kano.^63^ Briefly, the motion capture coordinates were exported as a CSV file using the Nexus software (version 2.14, VICON). The data was then screened by the pipeline to correct for mislabeling of the individuals’ ID and marker positions. The relative positions of eyes and beak to the attached head markers were reconstructed using a custom Python computer vision model. From the positions of eyes and beak, the head local coordinate system was reconstructed with the midpoint of the two eyes as the origin and the Y axis pointing to the horizon (defined as 30° above the origin-beak line in elevation). The reconstructed head was then applied onto the 3D coordinates tracked by the motion capture system to define the head local coordinate system during the whole trial, then filtered and smoothed to eliminate noise.

#### Definition of looking

From the head local coordinate system, we reconstructed the visual field of the individuals. As in a previous studies,^49^ foveal projections were defined as visual cones with their center lines projecting ±75° in azimuth and 0° in elevation in the head’s local coordinate system, with 10° error margin to accommodate for eye movement and noise.

Consistent with previous studies, our pigeons primarily used one of their foveas to look at the objects in this study. Interestingly, they occasionally also used their binocular field around horizon, consistent with the previous observation that they use this region when attending to slow-moving objects or when perching.^53^^,^^54^ Notably, we observed no use of the lower-frontal visual field, which is typically employed to attend to close ground objects such as grains.^50^^,^^66^ This aligns with existing literature suggesting that pigeons are likely myopic in this region of their visual field.^67^^,^^68^ We included both foveal and binocular field use into the analysis, with the latter defined as a cone with its center line projecting 0° in both azimuth and elevation Figure 1D. We observed that pigeons used their foveas and binary visual fields to look at the moving object in 92% and 8% of the cases respectively.

The region of interest for target or distractor object was defined as a sphere of 50 cm diameter encompassing the window’s 30 cm width, with a 10 cm margin on each side. Looking was defined when at least one of its reconstructed gaze cones crossed the spheres. Several filters were applied to reduce random gaze crossings. First, we excluded frames in which the individual was making head saccades, either because the viewed object could be affected by motion blur or because visual processing may be inhibited during these movements in birds^69^ (note that this filter does not apply to head bobbing, during which vision is not inhibited^70^), and also when the individual was grooming, as the individual was likely not attending to the environment. Grooming was automatically detected using a predefined algorithm.^49^ Second, we only considered fixations longer than 300 ms onto the object. Third, to ensure that only relevant demonstrator looks were considered for each observer, demonstrator looks were excluded when the observer was grooming.

#### Data exclusion

Pigeons that flew off or switched tables during the presentation, and test presentations where no demonstrator looked at the target were excluded from the analysis (Exp. 1: 7 of 842 observations; Exp. 2: 8 of 288 observations; Exp. 3: 20 of 690 observations).

#### Statistical analysis

We fitted the data with generalized linear mixed models (GLMMs) in R.^71^ The main response variables were the likelihood of an observer looking at either the target or distractor location (analyzed using binomial models) and the number of looks at the target or distractor location per presentation (analyzed using Poisson models, number of looks was considered count data and the distribution was confirmed by the absence of overdispersion). The main predictor were the condition (test or control) to test the presence of gaze following in each experiment and the “actual number of demonstrators” to test the effect of a collective effect on gaze following. The latter variable represented the number of pigeons on the demonstrator side that gave a gaze cue, i.e., that looked at the target at least once during the presentation. We have also tried the average number of demonstrator looking at any given time as predictors for the collective effect, but as it yielded the same results as only using the actual number of demonstrators, we did not include it in the results. For all models, we also included control predictors, i.e., the trial number, the presentation number, the presentation location (for Exp. 1 and 3, as Exp. 2 only had the top location), the experiment number (1 or 3, only for the combined analysis), the random effect of the individual ID, and random slopes for all fixed effects. Although the sex of the individual was not included (to simplify the models, as it was not our main question) this effect was accounted for in the random effect of the individual ID. Additionally, we confirmed the absence of influential individuals that might have dragged the results by examining the Cook’s distance.

For all models, all numerical variables were normalized to control for scale effects. Detailed R formulas are provided in Table S1. In addition, we checked the collinearity of the explanatory variables in all models, and for Poisson models, we checked for overdispersion. Model details, including the number of observations (each representing data from one observer pigeon during a single object presentation) as well as the detailed results for the control variables can be found in Table S2.
