# Supplementary material for: Gaze following in pigeons increases with the number of demonstrators
Source: iScience. 2025 Jun 9;28(7):112857. doi: 10.1016/j.isci.2025.112857 (PMC12221642; doi:10.1016/j.isci.2025.112857)
Supplement: Document S1. Figures S1–S3 and Tables S1 and S2 [file mmc1.pdf]

iScience, Volume *28*

Supplemental information

Gaze following in pigeons increases with the number of demonstrators

Mathilde Delacoux, Akihiro Itahara, and Fumihiro Kano

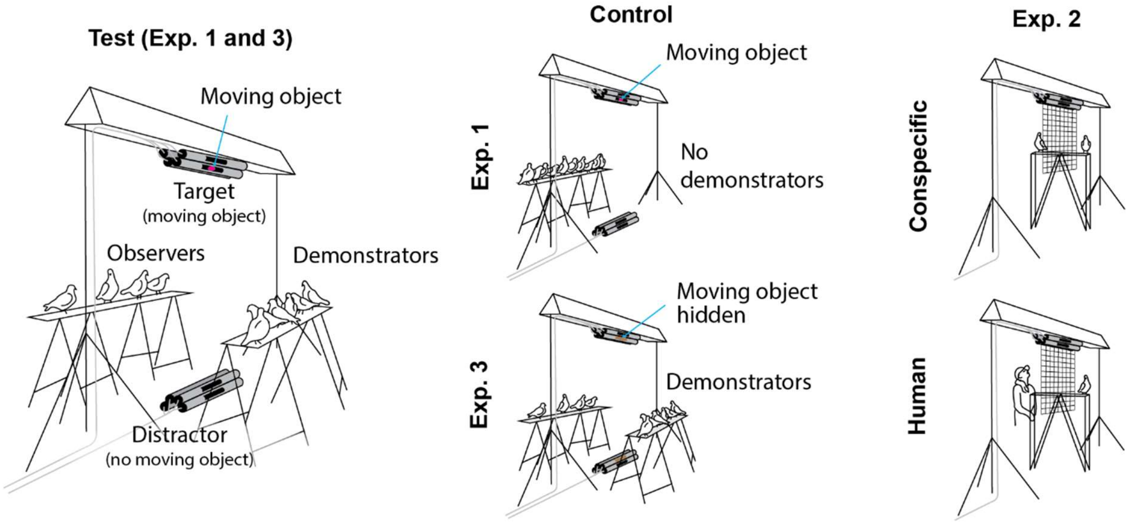

Figure S1: Different setup used in the study. Exp. 1 and 3 were conducted in a group setting, and the test conditions are identical in both, but the control differed. For Exp. 1, all 10 pigeons were moved to the observer side in such a way that there was no demonstrators. For Exp. 3, the windows from some of the tubes were occluded; there were demonstrators but they could not see the moving object and do not give a gaze cue. After conducting the first set of group experiments (Exp. 1), and to show the presence of gaze following in pigeons in a "test vs. control" condition design, we tested our pigeons in a more traditional setup (Exp. 2 and Exp. 2 follow-up). We ran a few tests in a dyadic setting, where the behavior of a focal subject (observer) was quantified in the presence of one demonstrator giving a gaze cue. We first used a conspecific demonstrator (Exp. 2), then a human demonstrator (Exp. 2 follow-up). Each approach had advantages: whereas a conspecific's gaze cue was expected to provide more biologically relevant information, a human demonstrator could give a more standardized cue in a more controlled manner. The latter aspect was particularly important, as demonstrator pigeons started to habituate to the moving objects in the tube, and no longer exhibited clear gaze cues in the later trials. It is worth noting that, for Exp. 2, a preliminary attempt failed because the object was too low and the pigeon's gaze was inadvertently hitting the target, increasing the noise. As a result, this experiment had to be rerun with a higher object.

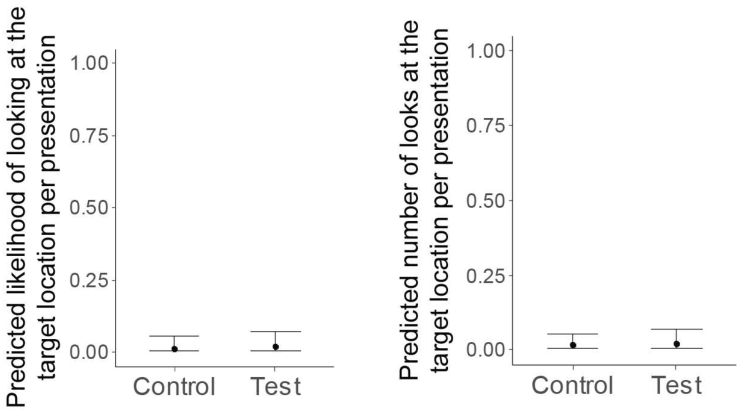

Figure S2: Results of the human demonstrator dyad experiment. Test-control difference in the likelihood of looking at the target location and the number of looks at the target location for Exp. 2 follow-up (human demonstrator).

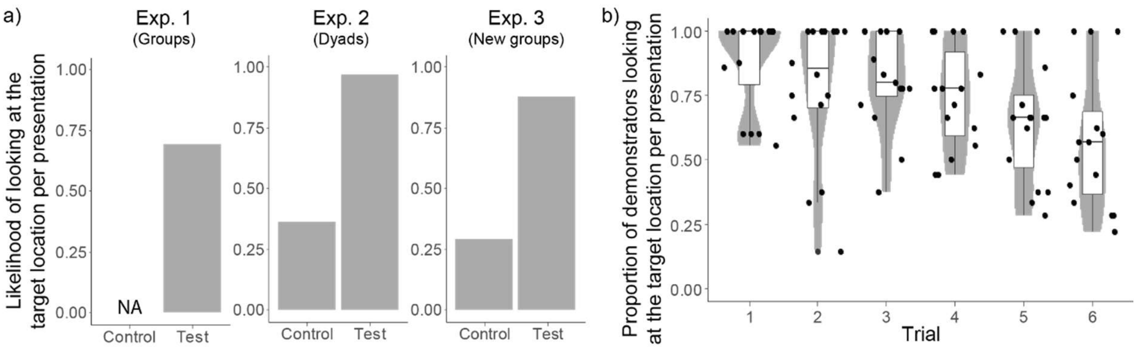

Figure S3: Demonstrators' response to the moving objects. To ascertain that the demonstrators' looking behavior was different in the test and control conditions, we extracted the number of demonstrators that gave a gaze cue in both conditions. a) Likelihood of the demonstrators looking at the target location during the test (demonstrators can see the moving object) and control (demonstrators cannot see the moving object) presentations for each experiment. In the Test condition of Exp. 1, 69.4% of the demonstrators looked at the target location at least once in the presentation. Note that for Exp. 1, there was no demonstrator in the control condition, hence the number of "actual" demonstrators cannot be extracted in the control condition. In the dyadic setup (Exp. 2), the demonstrator looked in 97.3% of the test conditions (with a visible moving object) and 36.4% in the control condition (the object is moving but not visible). In Exp. 3, 86.3% of the demonstrators looked at the target object during test presentations against 29.2% during control. This shows that the demonstrators were more likely to look at the object when they could see the moving object compared to when they could not see it. b) Evolution of the proportion of pigeons on the demonstrator table that looked at the object at least once during the presentation over the course of the trials. The lower percentage of looks in Exp. 1 compared to Exp. 2 and 3 is likely related to a decrease of the demonstrators' response over the course of the trials related to habituation. The distribution is represented using a violin plot (grey shade) and a boxplot (the boxes represent the 0.25 and 0.75 quartiles, with the median represented as a line inside the box, and the whiskers the minimum and maximum values within the lower/upper quartile ± 1.5 times the interquartile range), and the raw observations are displayed with a horizontal jitter for better visualization.

Table S1: R formulas used for the different models. The models from question 1) were ran with the datasets from Exp. 1, Exp. 2, Exp. 2 follow-up and Exp. 3 (note that for Exp. 2 and Exp. 2 follow-up, the Presentation_location was not included as only the top object was used). The models from question 4) were ran with the dataset from Exp. 3 only as only this dataset showed a significant Test-Control difference. The first 2 models from question 2) were ran with the test data from Exp. 1 and Exp. 3 as well as both datasets combined (note that the Experiment number variable was removed when testing the datasets separately). For the combined dataset, we also tested an alternative model using the Nb_demo (number of demonstrators standing on the table) instead of Nb_actual_demo (number of demonstrators that looked at the target location) to assess the importance of the looking behavior of the demonstrators. Finally, the models from question 3) were ran using the combined dataset to check for the presence of a non-linear effect (saturation).

	Question	R formula
Gaze following in pigeons	1) Comparison test-control	glmer(Looked_or_not ~ Condition + scale(Trial_nb) + scale(Presentation_nb) + scale(Presentation_location) + (Condition + scale(Trial_nb) + scale(Presentation_nb) + scale(Presentation_location) | Individual_ID), Data, family = binomial(link = "logit"))
		glmer(Nb_looks ~ Condition + scale(Trial_nb) + scale(Presentation_nb) + scale(Presentation_location) + (Condition + scale(Trial_nb) + scale(Presentation_nb) + scale(Presentation_location) | Individual_ID), Data, family = poisson(link = "log"))
	4) Comparison target-distractor	glmer(Firstlook_is_target ~ Condition + scale(Trial_nb) + scale(Presentation_nb) + scale(Presentation_location) + (Condition + scale(Trial_nb) + scale(Presentation_nb) + scale(Presentation_location) | Individual_ID), Data, family = binomial(link = "logit"))
		glmer(Nb_looks ~ Object * Condition + scale(Trial_nb) + scale(Presentation_nb) + scale(Presentation_location) + (Object * Condition + scale(Trial_nb) + scale(Presentation_nb) + scale(Presentation_location) | Individual_ID), Data, family = poisson(link = "log"))
		glmer(Looked_or_not ~ Object * Condition + scale(Trial_nb) + scale(Presentation_nb) + scale(Presentation_location) + (Object * Condition + scale(Trial_nb) + scale(Presentation_nb) + scale(Presentation_location) | Individual_ID), Data, family = binomial(link = "logit"))
Collective effect	2) Collective effect	glmer(Looked_or_not ~ scale(Nb_actual_demo) + scale(Experiment) + scale(Trial_nb) + scale(Presentation_nb) + scale(Presentation_location) + (scale(Nb_actual_demo) + scale(Experiment) + scale(Trial_nb) + scale(Presentation_nb) + scale(Presentation_location) | Individual_ID), Data, family = binomial(link = "logit"))
		glmer(Nb_looks ~ scale(Nb_actual_demo) + scale(Experiment) + scale(Trial_nb) + scale(Presentation_nb) + scale(Presentation_location) + (scale(Nb_actual_demo) + scale(Experiment) + scale(Trial_nb) + scale(Presentation_nb) + scale(Presentation_location) | Individual_ID), Data, family = poisson(link = "log"))
		glmer(Looked_or_not ~ scale(Nb_demo) + scale(Experiment) + scale(Trial_nb) + scale(Presentation_nb) + scale(Presentation_location) + (scale(Nb_demo) + scale(Experiment) + scale(Trial_nb) + scale(Presentation_nb) + scale(Presentation_location) | Individual_ID), Data, family = binomial(link = "logit"))
		glmer(Nb_looks ~ scale(Nb_demo) + scale(Experiment) + scale(Trial_nb) + scale(Presentation_nb) + scale(Presentation_location) + (scale(Nb_demo) + scale(Experiment) + scale(Trial_nb) + scale(Presentation_nb) + scale(Presentation_location) | Individual_ID), Data, family = poisson(link = "log"))
	3) Non-linear collective effect	glmer(Looked_or_not ~ scale(Nb_actual_demo) + I(scale(Nb_actual_demo^2)) + scale(Experiment) + scale(Trial_nb) + scale(Presentation_nb) + scale(Presentation_location) + (scale(Nb_actual_demo) + I(scale(Nb_actual_demo^2)) + scale(Experiment) + scale(Trial_nb) + scale(Presentation_nb) + scale(Presentation_location) | Individual_ID), Data, family = binomial(link = "logit"))
		glmer(Nb_looks ~ scale(Nb_actual_demo) + I(scale(Nb_actual_demo^2)) + scale(Experiment) + scale(Trial_nb) + scale(Presentation_nb) + scale(Presentation_location) + (scale(Nb_actual_demo) + I(scale(Nb_actual_demo^2)) + scale(Experiment) + scale(Trial_nb) + scale(Presentation_nb) + scale(Presentation_location) | Individual_ID), Data, family = poisson(link = "log"))

Table S2: Model results presenting the detailed information for each predictor in every model used in this study. It includes the beta estimates from the model, as well as the Chi-square value, degrees of freedom, and p-values derived from the likelihood ratio test. Note that the AICs are only comparable for models using the same response, distribution and dataset. Hence, AICs were only provided for models that have at least one other model they can be compared with.

	Question	Exp.	Response	Type	Variable	# of obs.	Beta	Chi-sq	Df	p	AIC
Gaze following in pigeons	1) Comparison test-control	Exp. 1	likelihood of looking	Test variable	Condition (Test)	835	-0.066	0.113	1	0.73631	-
				Control variable	Trial #	835	-0.123	1.125	1	0.28892	
					Presentation #	835	-0.103	0.756	1	0.38461	
					Presentation location (Top)	835	-0.694	19.015	1	0.00001	
		Exp. 1	# of looks	Test variable	Condition (Test)	835	0.061	0.077	1	0.78205	-
				Control variable	Trial #	835	-0.050	0.179	1	0.67246	
					Presentation #	835	-0.021	0.041	1	0.83947	
					Presentation location (Top)	835	-0.781	20.381	1	0.00001	
		Exp 2	likelihood of looking	Test variable	Condition (Control)	280	-0.443	1.848	1	0.17403	-
				Control variable	Trial #	280	-0.030	0.035	1	0.85217	
					Presentation #	280	-0.045	0.063	1	0.80111	
		Exp 2	# of looks	Test variable	Condition (Control)	280	-0.335	2.006	1	0.15673	-
				Control variable	Trial #	280	-0.141	0.901	1	0.34244	
					Presentation #	280	-0.106	0.510	1	0.47518	
		Exp 2 (follow-up)	likelihood of looking	Test variable	Condition (Control)	288	-0.393	0.035	1	0.85169	-
				Control variable	Trial #	288	0.003	-0.042	1	1	
					Presentation #	288	-0.200	0.122	1	0.72715	
		Exp 2 (follow-up)	# of looks	Test variable	Condition (Control)	288	-0.250	0.086	1	0.76941	-
				Control variable	Trial #	288	0.214	0.264	1	0.60732	
					Presentation #	288	-0.436	1.124	1	0.28916	
		Exp. 3	likelihood of looking	Test variable	Condition (Test)	670	0.448	4.964	1	0.02589	-
				Control variable	Trial #	670	0.061	0.256	1	0.61268	
					Presentation #	670	0.032	0.081	1	0.77595	
					Presentation location (Top)	670	-0.445	9.497	1	0.00206	
		Exp. 3	# of looks	Test variable	Condition (Test)	670	0.425	6.574	1	0.01035	-
				Control variable	Trial #	670	0.061	0.345	1	0.55674	
					Presentation #	670	0.095	1.060	1	0.30316	
					Presentation location (Top)	670	-0.503	14.836	1	0.00012	
		Exp. 3		Test variable	Condition (Test)	269	1.005	5.917	1	0.01499	-

			First look is target (binary)	Control variable	Trial #	269	-0.075	0.074	1	0.7854	
					Presentation #	269	-0.225	0.513	1	0.47375	
					Presentation location (Top)	269	-1.382	20.844	1	< 0.00001	
	4) Comparison target-distractor	Exp. 3	# of looks	Test variable	Condition (Test) * Object (Distr)	1340	-0.644	5.287	1	0.02149	-
				Control variable	Trial #	1340	-0.055	0.527	1	0.46807	
					Presentation #	1340	0.042	0.396	1	0.52906	
					Presentation location (Top)	1340	-0.087	1.177	1	0.27788	
		Exp. 3	likelihood of looking	Test variable	Condition (Test) * Object (Distr)	1340	-0.565	3.556	1	0.05931	-
				Control variable	Trial #	1340	-0.040	0.248	1	0.61834	
					Presentation #	1340	-0.002	-0.001	1	1	
					Presentation location (Top)	1340	-0.034	0.169	1	0.68109	
Collective effect	2) Collective effect	Exp. 1	likelihood of looking	Test variable	# of demo that looked	357	0.567	3.215	1	0.07296	-
				Control variable	Trial #	357	-0.453	2.572	1	0.10874	
					Presentation #	357	-0.924	13.647	1	0.00022	
					Presentation location (Top)	357	-0.406	2.293	1	0.12992	
		Exp. 1	# of looks	Test variable	# of demo that looked	357	0.583	6.028	1	0.01408	-
				Control variable	Trial #	357	-0.255	1.087	1	0.29723	
					Presentation #	357	-0.728	11.742	1	0.00061	
					Presentation location (Top)	357	-0.409	3.332	1	0.06794	
		Exp. 3	likelihood of looking	Test variable	# of demo that looked	318	0.183	0.213	1	0.64481	-
				Control variable	Trial #	318	0.247	1.009	1	0.31519	
					Presentation #	318	0.101	-0.320	1	1	
					Presentation location (Top)	318	-0.491	4.499	1	0.03391	
		Exp. 3	# of looks	Test variable	# of demo that looked	318	0.263	4.535	1	0.03321	-
				Control variable	Trial #	318	0.226	2.507	1	0.11331	
					Presentation #	318	0.134	1.088	1	0.29698	
					Presentation location (Top)	318	-0.425	6.428	1	0.01123	
		Combined (Exp. 1 + 3)	likelihood of looking	Test variable	# of demo that looked	675	0.285	3.623	1	0.05699	685.90
				Control variable	Experiment	675	0.380	5.304	1	0.02128	
					Trial #	675	-0.089	0.283	1	0.59464	
					Presentation #	675	-0.224	2.624	1	0.10524	
					Presentation location (Top)	675	-0.495	10.467	1	0.00122	
		Combined (Exp. 1 + 3)	# of looks	Test variable	# of demo that looked	675	0.324	8.902	1	0.00285	1050.90
				Control variable	Experiment	675	0.312	3.923	1	0.04762	
					Trial #	675	0.082	0.611	1	0.43445	

				Presentation #	675	-0.101	1.241	1	0.2652	
				Presentation location (Top)	675	-0.463	12.657	1	0.00037	
	Combined (Exp. 1 + 3)	likelihood of looking	Test variable	# of demo on the other table	675	0.221	2.464	1	0.11651	690.46
			Control variable	Experiment	675	0.453	8.487	1	0.00358	
				Trial #	675	-0.120	0.506	1	0.47693	
				Presentation #	675	-0.244	3.324	1	0.06827	
				Presentation location (Top)	675	-0.474	9.549	1	0.002	
	Combined (Exp. 1 + 3)	# of looks	Test variable	# of demo on the other table	675	0.207	3.893	1	0.04848	1058.35
			Control variable	Experiment	675	0.434	7.327	1	0.00679	
				Trial #	675	0.068	0.188	1	0.66477	
				Presentation #	675	-0.098	0.925	1	0.33626	
				Presentation location (Top)	675	-0.473	12.834	1	0.00034	
3) Non-linear collective effect	Combined (Exp. 1 + 3)	likelihood of looking	Test variable	# of demo that looked	675	0.925	2.683	1	0.1014	696.95
				# of demo that looked squared	675	-0.646	1.504	1	0.21999	
			Control variable	Experiment	675	0.410	6.503	1	0.01077	
				Trial #	675	-0.103	0.373	1	0.54154	
				Presentation #	675	-0.257	3.122	1	0.07723	
				Presentation location (Top)	675	-0.498	10.441	1	0.00123	
	Combined (Exp. 1 + 3)	# of looks	Test variable	# of demo that looked	675	0.837	3.185	1	0.07434	1057.01
				# of demo that looked squared	675	-0.556	1.478	1	0.22415	
			Control variable	Experiment	675	0.344	4.605	1	0.03188	
				Trial #	675	0.056	0.134	1	0.71404	
				Presentation #	675	-0.119	1.113	1	0.29139	
				Presentation location (Top)	675	-0.443	12.138	1	0.00049	
